# Equilibrium Conformation of a Novel Cable-Driven Snake-Arm Robot under External Loads

**DOI:** 10.3390/mi13071149

**Published:** 2022-07-20

**Authors:** Long Huang, Bei Liu, Leiyu Zhang, Lairong Yin

**Affiliations:** 1 School of Automotive and Mechanical Engineering, Changsha University of Science and Technology, Changsha 410114, China; huanglongin@foxmail.com (L.H.); liubei1224@foxmail.com (B.L.); 2 Hunan Provincial Key Laboratory of Intelligent Manufacturing Technology for High-Performance Mechanical Equipment, Changsha University of Science and Technology, Changsha 410114, China; 3 College of Mechanical Engineering and Applied Electronics Technology, Beijing University of Technology, Beijing 100022, China; zhangleiyu1988@126.com

**Keywords:** cable-driven snake-arm robot, kinematics, workspace, static model, anti-parallelogram mechanism

## Abstract

Based on the anti-parallelogram mechanism, an approximate cylindrical rolling joint is proposed to develop a novel cable-driven snake-arm robot with multiple degrees of freedom (DOF). Furthermore, the kinematics of the cable-driven snake-arm robot are established, and the mapping between actuator space and joint space is simplified by bending decoupling motion in the multiple segments. The workspace and bending configurations of the robot are obtained. The static model is established by the principle of minimum potential energy. Furthermore, the simplified cable constraints in the static model are proposed through Taylor expansion, which facilitates the equilibrium conformation analysis of the robot under different external forces. The cable-driven snake-arm robot prototype is developed to verify the feasibility of the robot design and the availability of the static model through the experiments of the free bending motion and the external load on the robot.

## 1. Introduction

The cable-driven snake-arm robot has been widely used in different fields, such as minimally invasive surgery [1,2], industrial inspection [3,4], and field rescue [5] in confined environments, since it has significant advantages because of its multi-DOF bending motion. Each segment of the cable-driven snake-arm robot is mainly composed of several identical 1-DOF joints or 2-DOF joints connected in series by multi-side cables. The number of joints usually is significantly greater than the number of motors. Therefore, passive compliance may exist in the cable-driven snake-arm robot [6], which will result in the robot configuration being not controllable when the end of the robot is subject to external forces.

To address this problem, Kim et al. presented an adjusting-stiffness cable-driven hyper-redundant robot with several 1-DOF cylindrical rolling joints, and the method to avoid the passive compliance is proposed through the positive sum of the cable length changes [7]. Based on gears and constraint links, Hwang et al. proposed a novel cable-driven snake-arm robot and validated that there is no slack in the cables when the joint bends to an arbitrary configuration [8]. Kim et al. presented a novel 1-DOF rolling joint with block mechanisms and discussed the effect between passive compliance and cable tension [9]. Suh et al. designed a cable-driven snake-arm robot including rolling joints with elastic fixtures, which improves the stability of the robot [10]. Compared with regular serial mechanisms, the parallel link mechanism has better flexibility [11,12,13]. Based on the link mechanism, Shin et al. designed an approximate rolling joint to ensure that the cables on both sides are always tensioned [14]. Based on the parallel isosceles trapezoidal-link mechanism, Zhang et al. proposed a reconfigurable joint to develop a cable-driven snake-arm robot prototype without cable slack [15].

However, the bending motion in all directions of most of the prior cable-driven snake-arm robots is usually coupled. That means the rotation output of the robot is controlled by multiple pairs of antagonistic cable inputs in each segment [15,16,17], which results in the mapping between the actuator space and joint space becoming more complex. Wu et al. realized the decoupling motion of the proximal end and distal wrist joint in a continuum robot by a reverse bi-continuum mechanism [18]. Based on the cable sheath, Phee et al. avoided the coupling motion in the cable-driven snake-arm robot. In addition, when the cable-driven snake-arm robot works in complex environments, it will inevitably be subjected to external forces that make the robot configuration change [19]. Therefore, in order to evaluate the equilibrium conformation, it is essential to establish the static model of the snake-arm robot under external loads.

Similar to octopus tentacles [20], elephant trunks [21], and tongues [22,23], continuum robots can achieve continuous and smooth multi-DOF bending motion by their elastic deformation [24,25]. By contrast, the biggest difference of the cable-driven snake-arm robot is that it contains several identical rigid discrete joints, while the continuum robot does not have rigid discrete joints. Due to the discreteness, the prior static models are not suitable for a cable-driven snake-arm robot with multiple discrete joints. In recent years, many researchers have studied the static model suitable for cable-driven continuum robots without discrete joints but not for cable-driven snake-arm robots. Based on the Cosserat rod method, Alqumsan et al. derived the static model of a continuous robot under external force [26]. Xu et al. established the static model of the continuum robot based on the elliptic integral method, which derives the mapping between the driving force and end position [27]. Considering gravity, the external force, and cable deformation, Yang et al. established the static model of the continuum robot according to the virtual work principle [28]. Venkiteswaran et al. presented the static model based on the pseudo-rigid body model, which is suitable for the non-constant curvature curved motion of the continuum robot [29]. However, these methods are established based on the material constitutive model, which cannot be directly applied to the snake-arm robot with discrete joints [30].

For the cable-driven snake-arm robot with discrete joints, Dong et al. calculated the rotational angle of each segment based on the static equilibrium equation, which predicts the robot configuration change under external forces [3]. Li et al. established the static equilibrium equation based on the Newton–Euler method and obtained the mapping between joint deformation and external forces [31]. Wang et al. also utilized the Newton–Euler method to establish the mapping between cable tension and joint deformation [32]. Kato et al. established the relationship between the cable tension and kinematics to predict the robot configuration [33]. Chen et al. proposed a new prediction method of the soft arm shape by combining the constant curvature model with the Euler–Bernoulli beam [34]. Although the static model can be established utilizing many methods, it is difficult to derive the equilibrium conformation of cable-driven snake-arm robots with complicated cable constraints. 

This paper proposes a novel cable-driven snake-arm robot with approximate cylindrical rolling joints based on anti-parallelogram mechanisms, which can avoid passive compliance. The decoupling bending motion can be realized between multiple segments in the cable-driven snake-arm robot, which can simplify kinematics. Based on the principle of minimum potential energy, the static model of the robot is established. Furthermore, the simplified cable constraints in the static model are proposed through Taylor expansion, which facilitates the equilibrium conformation analysis of the robot under different external forces, different spring stiffness, and different initial heights. Finally, the robot feasibility and the validity of the static model are verified by experiments.

## 2. Robot Design

### 2.1. Joint Design

**Figure 1:**
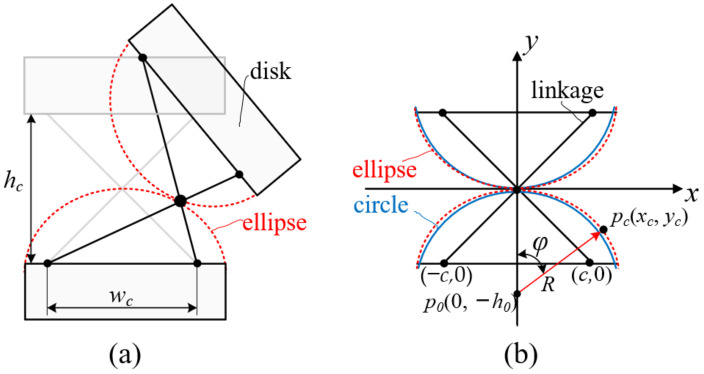
Sketch of anti-parallelogram mechanism. (**a**) The anti-parallelogram joint sketch. (**b**) The initial configuration of the anti-parallelogram joint.

According to the references [35,36,37,38,39], the rotation of the anti-parallelogram mechanism can be regarded as the pure rolling motion of two ellipses, as shown in Figure 1, and the hinge points between linkages and disks are regarded as the focus of the ellipse located at the major axis in an ellipse.

**Figure 2:**
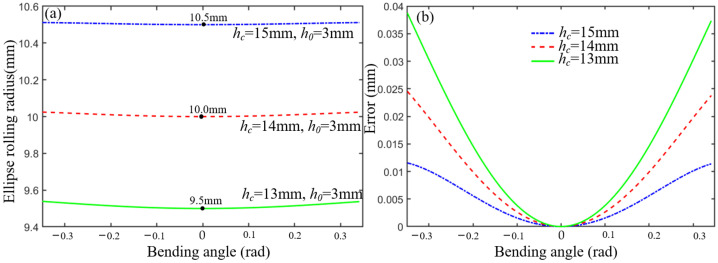
The influence of *h_c_* on *R*_0._ (**a**) The relationship between the *h_c_* and ellipse rolling radius. (**b**) The error analysis between *R*_0_ and *R* in different *h_c_*.

**Figure 3:**
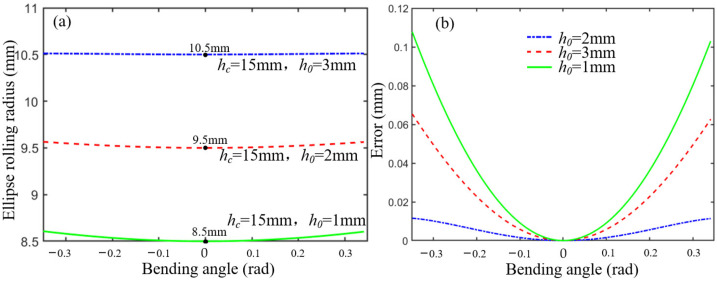
The influence of *h*_0_ on *R*_0._ (**a**) The relationship between the *h*_0_ and ellipse rolling radius. (**b**) The error analysis between *R*_0_ and *R* in different *h*_0_.

The focus coordinates are respectively (−*w_c_*/2, 0) and (*w_c_*/2, 0), while the length of the minor axis can be calculated by *b* = *h_c_*/2. Therefore, the ellipse equation can be expressed as

(1) $$\frac{x_{c}{}^{2}}{{\left(\frac{h_{c}}{2}\right)}^{2}+{\left(\frac{w_{c}}{2}\right)}^{2}}+\frac{y_{c}{}^{2}}{{\left(\frac{h_{c}}{2}\right)}^{2}}=1$$

where *h_c_* represents the distance between the two major axes of two adjacent ellipses and *w_c_* represents the distance between the two focuses of the ellipse. Assuming that there is a circle that approximates the ellipse, as shown in Figure 1b, *h*_0_ represents the distance between the circle center *p*_0_ (0, *h*_0_) and the origin. *y_c_* denotes the *y*-direction distance between the tangent point *p_c_* (*x_c_*, *y_c_*) of two ellipses and the center point *p*_0_, which can be calculated as

(2) $$y_{c}=\frac{1}{\tan\varphi }x_{c}-h_{0}$$

where $x_{c}=R_{0}\sin\varphi$. *R*_0_ represents the ellipse rolling radius, *φ* represents the rotation angle, and *h*_0_ represents the distance from the circle center to the origin. According to Equations (1) and (2), *R*_0_ can be obtained as

(3) $$R_{0}=\frac{h_{0}+\sqrt{h_{0}{}^{2}+\left(\frac{h_{c}{}^{2}}{4}+\frac{M}{\tan^{2}\varphi }\right)\cdot \left(\frac{h_{c}{}^{2}}{4}-h_{0}{}^{2}\right)\cdot \frac{\tan^{2}\varphi }{M}}}{\sin\varphi \cdot \left(\frac{h_{c}{}^{2}}{4}+\frac{M}{\tan^{2}\varphi }\right)\cdot \frac{\tan\varphi }{M}}$$

where sin *φ* is always positive due to the limitation of the maximum joint angle. When *w_c_* and *h*_0_ are known, *R*_0_ is only relevant to the initial joint height *h_c_* and rotation angle. The ellipse rolling motion can be approximated to cylindrical rolling motion by adjusting the structural parameters when *R*_0_ is very close to the constant *R*. The constant *R* can be regarded as a cylindrical rolling radius, and the difference between *R*_0_ and *R* can be defined as an error. Given that *h*_0_ = 3 mm and *w_c_* = 10 mm, the ellipse rolling radius *R*_0_ increases as *h_c_* increases, as shown in Figure 2a. The error decreases in the range of the bending angle, as shown in Figure 2b. Given that *h_c_* = 15 mm and *w_c_* = 10 mm, the ellipse rolling radius *R*_0_ increases as *h*_0_ increases, as shown in Figure 3a. The error decreases in the range of the bending angle, as shown in Figure 3b. Therefore, the error is less than 0.05 when *h_c_* = 15 mm and *h*_0_ = 3 mm.

**Figure 4:**
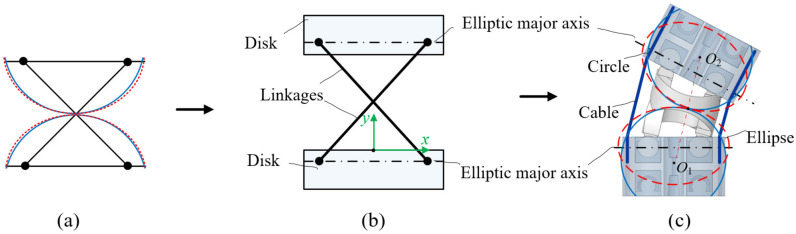
The joint structure design. (**a**) The initial anti-parallelogram mechanism sketch. (**b**) The initial joint sketch. (**c**) The joint design with anti-parallelogram mechanism.

Considering the working requirements of the cable-driven snake-arm robot, the following design principles should be simultaneously satisfied to propose the approximately cylindrical rolling joint. Firstly, the virtual rolling radius of the ellipse should be approximately a constant. Secondly, passive compliance should be avoided for approximate rolling joints to ensure that the snake-arm robot is controllable when its end is subject to external forces. Thirdly, the maximum bending angle of the rolling joint should not be less than 20 degrees. According to the anti-parallelogram mechanism, an approximate cylindrical rolling joint is designed, and each joint consists of two links and disks, as shown in Figure 4; the pivot of the links coincides with the major axis of two adjacent ellipses. 

In the rolling joint, the relationship between the cable length on both sides and the bending angle is derived as

(4) $$\begin{array}{l} l_{l}=2R-2\left(R-\frac{h_{c}+2h_{1}}{2}\right)\cos\frac{\psi }{2}-2r\sin\frac{\psi }{2}\\ l_{r}=2R-2\left(R-\frac{h_{c}+2h_{1}}{2}\right)\cos\frac{\psi }{2}+2r\sin\frac{\psi }{2} \end{array}$$

**Figure 5:**
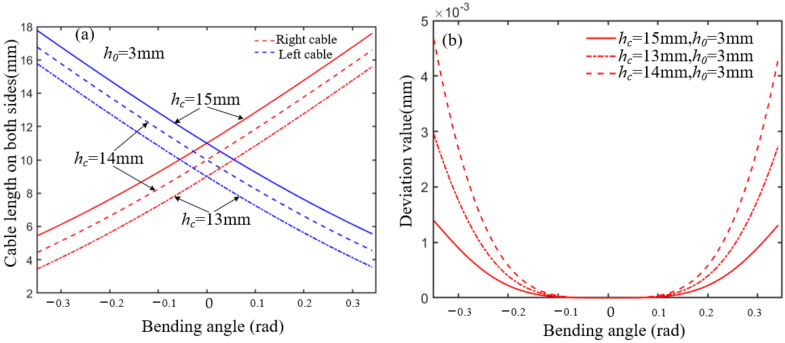
The cable length change on both sides and difference analysis. (**a**) The relationship between the *h_c_* and cable length on both sides. (**b**) The deviation value analysis between the *h_c_* and cable length in different *h_c_*.

Compared with the straight configuration of the joint, the length changes of the cable on both sides are obtained as

(5) $$\begin{array}{l} \Delta l_{l}=l_{l}\left(\varphi \right)-l_{l}\left(0\right)=2R_{1}-2\sqrt{R_{1}{}^{2}+r^{2}}\sin\left(\frac{\psi }{2}+\beta \right)\\ \Delta l_{r}=l_{r}\left(\varphi \right)-l_{r}\left(0\right)=2R_{1}+2\sqrt{R_{1}{}^{2}+r^{2}}\sin\left(\frac{\psi }{2}-\beta \right) \end{array}$$

where $R_{1}=R-\frac{h_{c}+2h_{1}}{2},\tan\beta =\frac{R_{1}}{r}$. *r* denotes the cable distribution circle radius. ψ denotes the joint bending angle. *h*_1_ denotes the distance between the major axis of the ellipse and the end surface of the disk. *h*_2_ denotes the disk thickness. *h*_3_ denotes the initial joint height. Therefore, given that *h*_0_ is 3 mm and *w_c_* is 10 mm, and *h_c_* = {13 mm, 14 mm, 15 mm}, the relationship between cable length and joint angle can be obtained by the ellipse rolling radius, as shown in Figure 5a. The difference between cable length on both sides, calculated by the ellipse rolling radius *R*_0_ and approximate cylindrical rolling radius *R*, will increase as *h_c_* increases in the range of the joint angles, as shown in Figure 5b.

**Figure 6:**
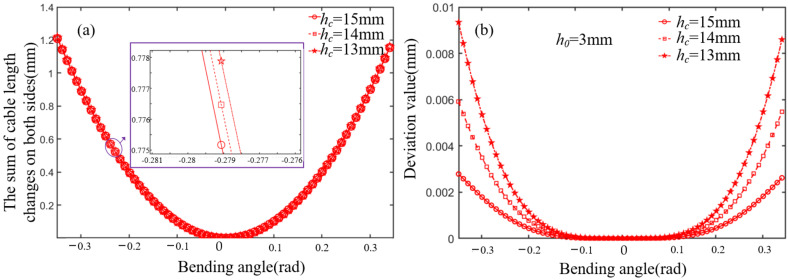
The sum of cable length change on both sides and difference analysis. (**a**) The relationship between the *h_c_* and the sum of cable length changes on both sides. (**b**) The deviation value analysis between the *h_c_* and the sum of cable length changes in different *h_c_*.

In addition, the sum of the cable length changes on both sides is always greater than zero, as shown in Figure 6a. The difference between cable length on both sides, calculated by the ellipse rolling radius *R*_0_ and approximate cylindrical rolling radius *R*, will decrease as *h_c_* increases in the range of the joint angles, as shown in Figure 6b. Therefore, the approximate cylindrical rolling radius *R* = 10.5 mm is preferred.

### 2.2. Manipulator Design

**Figure 7:**
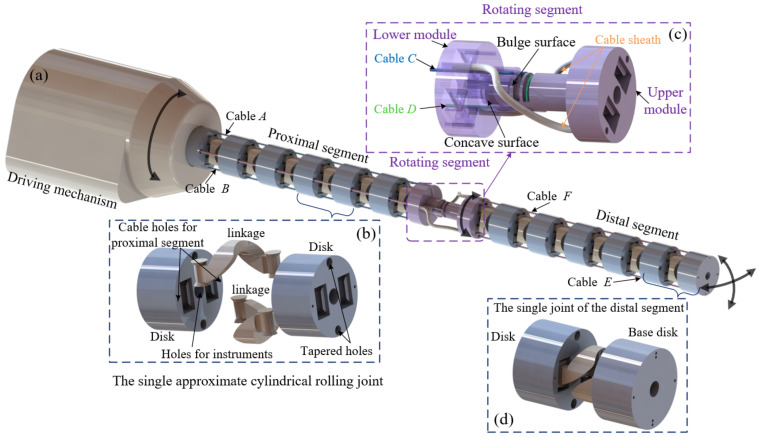
The CAD model of the cable-driven snake-arm robot. (**a**) The cable-driven snake-arm robot model. (**b**) The joint in the proximal segment. (**c**) The structure of the rotating segment. (**d**) The joint in the distal segment.

The cable-driven snake-arm robot is mainly composed of the manipulator and the driving mechanism, which can realize 4 DOF, as shown in Figure 7a. The range of rotating the driving mechanism is [−π, π]. Moreover, the manipulator contains a proximal segment, a rotating segment, and a distal segment. The rotating segment is located in the middle between the proximal and distal segments. The joint structure of the proximal segment is shown in Figure 7b. The specific structure of the rotating segment is shown in Figure 7c. The rotating angle range of the rotating segment is [−π/2, π/2]. The rotating segment comprises upper and lower modules (Figure 7c). The bulge surface of the upper module and the concave surface of the lower module cooperate to form one rotating pair; the antagonism always exists between the upper and lower modules to ensure that the rotating segment does not produce bending deformation. In addition, both ends of cables *C* and *D* are fixed in the upper module, respectively. The driving inputs of the rotating structure remain unchanged, that is, cables *C* and *D* do not change, which can ensure that the rotating segment does not produce torsional deformation and that the angle of the rotating segment will not affect the ability to hold the load in 3-dimensional space.

The joint structure of the distal segment is shown in Figure 7d. The proximal and distal segments of the robot are composed of several of the same approximately cylindrical rolling joints in series. Each joint contains two linkages and two disks, which can be regarded as the anti-parallelogram mechanism. When the linkage and disk interfere with each other, the maximum bending angle of the single joint can be reached. The maximum bending angle of the approximately cylindrical rolling joint is limited to 20°. According to the above design principles, the specific structure parameters of the robot are determined in Table 1.

The motion between the proximal segment, the rotating segment, and the distal segment is decoupling. Cables *A* and *B* are used to drive the proximal segment. Cables *C* and *D* are used to drive the rotating segment. Cables *E* and *F* are used to drive the distal segment. In the disk of the proximal segment, the cable hole for cable *C*, cable *D*, cable *E*, and cable *F* is a tapered hole, which ensures the cable length of cable *C*, cable *D*, cable *E*, and cable *F* is always equal to 2*R* when the proximal segment bends. The design difficulty of the driving mechanism is effectively reduced. The cable sheaths are reserved in the rotating segment to ensure the decoupling motion between the proximal and distal segments. In addition, the driving mechanism design of the proximal segment, the rotating segment, and the distal segment can be simplified according to the mapping between actuator space and joint space.

## 3. Kinematics

It is inevitable for the cable-driven snake-arm robots to establish the mapping among the actuator space, joint space, and task space to analyze the robot kinematics [40,41]. Through the bending decoupling motion, the mapping between the actuator space and joint space of the three segments is simplified to design the driving mechanism of the robot. The mapping between joint space and task space is established to analyze the workspace and different bending configurations of the novel cable-driven snake-arm robot.

### 3.1. The Mapping between Actuator Space and Task Space

The mappings between actuator space and joint space in the proximal segment, rotating segment, and distal segment are respectively established in this section. For the mapping between actuator space and joint space of the proximal segment, assume that the proximal segment contains *n* same cylindrical rolling joints. According to Equation (4), the relationship between the length of cables *A* and *B* and the bending angle $\psi_{1}$ can be expressed as

(6) $$\begin{array}{l} L_{Ap}=2nR-2n\left(R-\frac{h_{c}+2h_{1}}{2}\right)\cos\frac{\psi_{1}}{2}-2nr\sin\frac{\psi_{1}}{2}\\ L_{Bp}=2nR-2n\left(R-\frac{h_{c}+2h_{1}}{2}\right)\cos\frac{\psi_{1}}{2}+2nr\sin\frac{\psi_{1}}{2} \end{array}$$

where *L_Ap_* and *L_Bp_* represent, respectively, the relationship between the length of cables *A* and *B* and the bending angle.

Compared with the straight configuration, the relationship between cable length changes, and the angle $\psi_{1}$ in the bending configuration can be derived as

(7) $$\begin{array}{l} \Delta L_{Ap}=n\left(l_{l}\left(\psi_{1}\right)-l_{l}\left(0\right)\right)=2nR_{1}-2n\sqrt{R_{1}{}^{2}+r^{2}}\sin\left(\frac{\psi_{1}}{2}+\beta \right)\\ \Delta L_{Bp}=n\left(l_{r}\left(\psi_{1}\right)-l_{r}\left(0\right)\right)=2nR_{1}+2n\sqrt{R_{1}{}^{2}+r^{2}}\sin\left(\frac{\psi_{1}}{2}-\beta \right) \end{array}$$

where $\Delta L_{Ap}$ and $\Delta L_{Bp}$ represent, respectively, the relationship between the length changes of cables *A* and *B* and the bending angle. For the mapping between actuator space and joint space of the rotating segment, if the tapered cable holes are not reserved on disks of the proximal segment, the relationship between the length of cables *C* and *D* and the angle $\psi_{1}$ can be expressed as

(8) $$\begin{array}{l} \Delta L_{C}=\Delta L_{Cp}+\Delta L_{Cr}=-2nR_{1}\cos\frac{\psi_{1}}{2}+r_{s}\gamma \\ \Delta L_{D}=\Delta L_{Dp}+\Delta L_{Dr}=-2nR_{1}\cos\frac{\psi_{1}}{2}-r_{s}\gamma \end{array}$$

where $\Delta L_{Cp}=\Delta L_{Dp}=-2nR_{1}\cos\frac{\psi_{1}}{2}$ represents, respectively, the length changes of cables *C* and *D* and angle $\psi_{1}$. $\Delta L_{Cr}=r_{s}\gamma$ and $\Delta L_{Dr}=-r_{s}\gamma$ represent, respectively, the cable length changes of cables *C* and *D* and rotating angle *γ*. *r_s_* represents the radius of a rotating pulley in a rotating segment. However, if the tapered cable holes are reserved on disks of the proximal segment, cable length changes of cables *C* and *D* in the proximal segment are always equal to zero. Therefore, Equation (8) can be rewritten as

(9) $$\begin{array}{l} \Delta L_{Cp}=\Delta L_{Cp}+\Delta L_{Cr}=r_{s}\gamma \\ \Delta L_{Dp}=\Delta L_{Dp}+\Delta L_{Dr}=-r_{s}\gamma \end{array}$$

For the mapping between actuator space and joint space of the distal segment, the motion of the proximal and rotating segments will cause length changes of cables *E* and *F*. In the rotating segment, the cable sheaths of cables *E* and *F* are reserved to guarantee that the lengths of cables *E* and *F* are respectively equal to the cable sheath’s length. If the tapered cable holes are not reserved on disks of the proximal segment, the relationship between the cable length of the distal segment and angles can be expressed as

(10) $$\begin{array}{l} L_{E}=L_{Ed}+L_{Ep}+L_{r}=n\left(2R-2\left(R-\frac{h_{c}+2h_{1}}{2}\right)\cos\frac{\psi_{2}}{2}-2r\sin\frac{\psi_{2}}{2}\right)+n\left(2R-2\left(R-\frac{h_{c}}{2}\right)\cos\frac{\psi_{1}}{2}\right)+L_{r}\\ L_{F}=L_{Fd}+L_{Fp}+L_{r}=n\left(2R-2\left(R-\frac{h_{c}+2h_{1}}{2}\right)\cos\frac{\psi_{2}}{2}+2r\sin\frac{\psi_{2}}{2}\right)+n\left(2R-2\left(R-\frac{h_{c}}{2}\right)\cos\frac{\psi_{1}}{2}\right)+L_{r} \end{array}$$

where *L_E_* and *L_F_* represent, respectively, the relationship between the length of cables *E* and *F* and the bending angle $\psi_{2}$. *L_Ed_* and *L_Fd_* represent, respectively, the relationship between the length of cables *E* and *F* and the bending angle $\psi_{2}$ in the distal segment. *L_Ep_* and *L_Fp_* represent, respectively, the relationship between the length of cables *E* and *F* and the bending angle $\psi_{1}$ in the proximal segment. *L_r_* represents cable sheath length. If the tapered cable holes are reserved on disks of the proximal segment, length changes of cables *C* and *D* in the proximal segment are also always equal to zero. Therefore, Equation (10) can be rewritten as

(11) $$\begin{array}{l} \Delta L_{E}=-2nR_{1}+2n\sqrt{R_{1}{}^{2}+r^{2}}\sin\left(\frac{\psi_{2}}{2}-\beta \right)\\ \Delta L_{F}=-2nR_{1}+2n\sqrt{R_{1}{}^{2}+r^{2}}\sin\left(\frac{\psi_{2}}{2}+\beta \right) \end{array}$$

**Figure 8:**
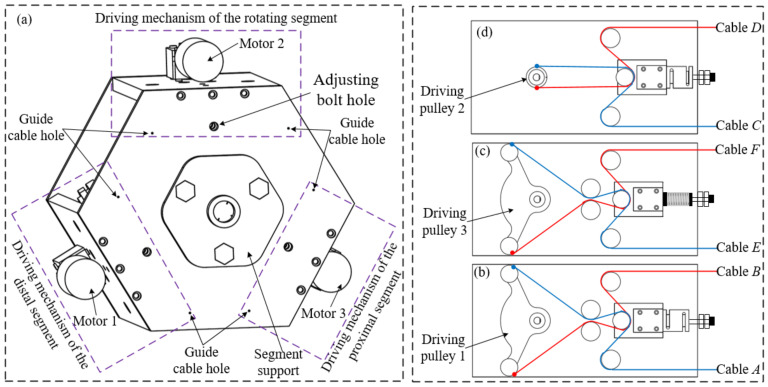
The driving mechanism of the robot. (**a**) The structure of the driving mechanism. (**b**) The driving mechanism of the rotating segment. (**c**) The driving mechanism of the distal segment. (**d**) The driving mechanism of the distal segment.

According to Equation (11), the decoupling bending performance can simplify the mapping between the actuator space and joint space of the distal segment. Since the expression form of the cable length function of cables *E* and *F* are the same as that of cables *A* and *B*, the driving mechanism of proximal and distal segments can be consistent. The mapping between the actuator space and joint space of the robot can be obtained by establishing the relationship between the length of cables and joint variables. Therefore, the driving mechanism design of the cable-driven snake-arm robot is shown in Figure 8.

### 3.2. The Mapping between Actuator Space and Task Space

**Figure 9:**
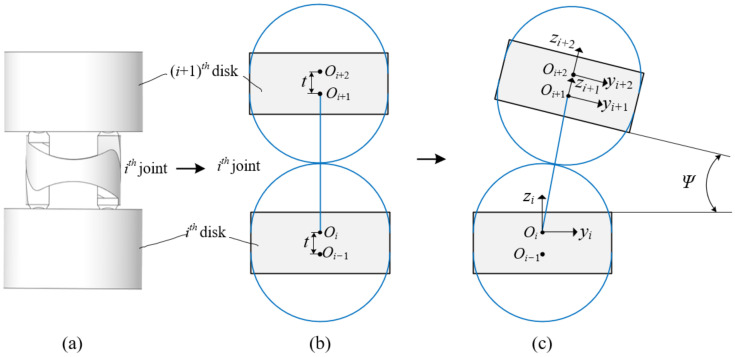
The joint coordinate system of a single joint. (**a**) The *i*th joint in the initial configuration. (**b**) The coordinate system in the initial configuration. (**c**) The coordinate system in the bending configuration.

In this section, the *i*th joint is considered as an example to establish the joint coordinate system, as shown in Figure 9. The coordinates {***O****_i_*}, {***O****_i_*_−1_}, {***O****_i_*_+1_}, and {***O****_i_*_+2_} are established at the center position of the circle. The ***z****_i_* axis is perpendicular to the surface of the *i*th disk, and the ***y****_i_* axis is parallel to the surface of the *i*th disk. The ***z****_i_*_+1_ axis is perpendicular to the surface of the (*i* + 1)th disk, and the ***y****_i_*_+1_ axis is parallel to the surface of the (*i* + 1)th disk. The coordinate system follows the right-hand rule.

The transformation process from the coordinate {***O****_i_*} to coordinate {***O****_i_*_+2_} are as follows. The coordinate {***O****_i_*_+1_} is obtained by the coordinate system {***O****_i_*} rotating ψ/2 about the ***x****_i_* axis, transferring 2*R* along the ***z*** axis, and rotating ψ/2 about the new ***x****_i_* axis. The coordinate {***O****_i_*_+2_} is obtained by the coordinate system {***O****_i_*_+1_} transferring *t* along the ***z****_i_*_+1_ axis.

**Figure 10:**
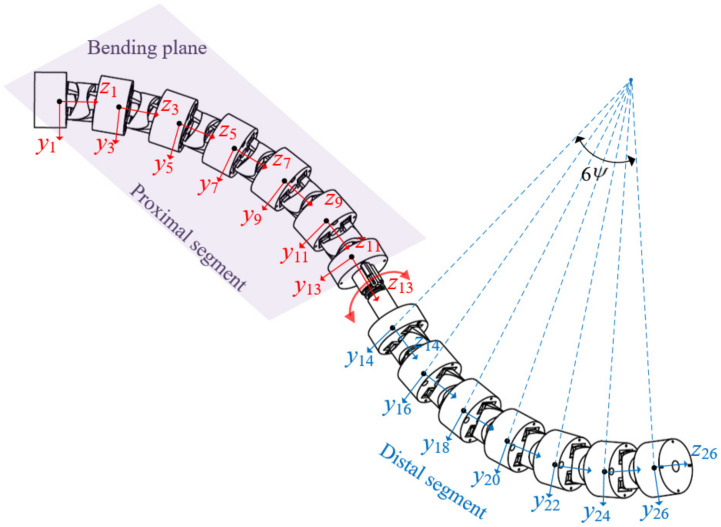
The robot coordinate system.

(12) Ti=rotx,ψ2transz,2Rrotx,ψ2transz,t

where Ti is the homogeneous transformation matrix from *i*th joint to (*i* + 1)th joint, and *t* is the distance between adjacent circle centers. Based on the joint kinematics, the mapping between the joint space and task space of the cable-driven snake-arm robot is established to analyze the workspace and different bending configurations, as shown in Figure 10.

According to Equation (12), the homogeneous transformation matrix from coordinate {***O***_1_} to {***O***_13_} can be obtained as

(13) T113=rotz,α⋅Tii+16ψ1⋅transz,h0−h1

where *α*∈[−π, π] represents the rotating angle of the driving mechanism. According to the geometric structure, the end position of the proximal segment can be expressed as

(14) p113=pxpypz=2R+tcosα∑i=16sin2i−12ψ12R+tsinα∑i=16sin2i−12ψ12R+t∑i=16cos2i−12ψ1

According to Equation (13), the homogeneous transformation matrix from coordinate {***O***_1_} to {***O***_26_} can be obtained as

(15) T126=T113φ1⋅transz,h3⋅T1426

where $\mathrm{trans}\left(z,h_{3}\right)$ is obtained by the coordinate {***O***_13_} transferring the height of the rotating segment, and T1426 represents the homogeneous transformation matrix from coordinate {***O***_14_} to coordinate {***O***_26_}, which can be expressed as

**Figure 11:**
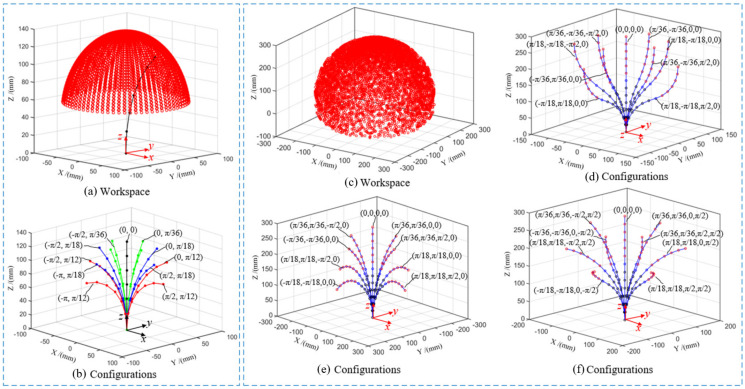
The workspace and bending configurations of the robot. (**a**) The workspace of the proximal segment. (**b**) The configurations of the proximal segment. (**c**) The workspace of the robot. (**d**) The bending configuration in different direction. (**e**) The bending configuration in same direction. (**f**) The bending configuration in different bending angles.

(16) T1426=rotz,γ⋅Tii+16ψ2

where *γ*∈[−π/2, π/2] is the rotating angle of the rotating segment. Therefore, the mapping between joint space and task space of the cable-driven snake-arm robot can be established by Equations (12)–(16). The direction angle α∈{0, ±π/2, π} and bending angles ψ∈{0, π/36, π/18, π/12} are selected to obtain the workspace and bending configuration of every segment of the robot. The workspace and bending configuration of the proximal segment are shown in Figure 11a,b, and the workspace and bending configuration of the robot are shown in Figure 11c–f.

## 4. Equilibrium Conformations Analysis under External Loads

### 4.1. Static Model

**Figure 12:**
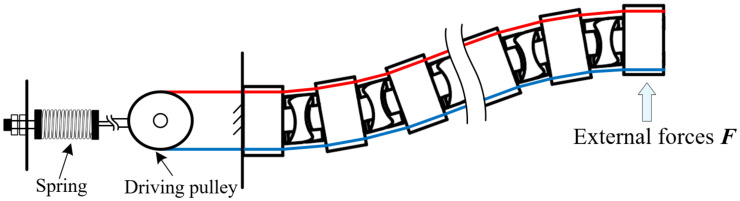
The static model of the distal segment in the cable-driven snake-arm robot.

According to Figure 8, when the end of the distal segment is subject to external forces, and the inputs of the driving pulley remain constant, the configuration of the distal segment changes from the straight configuration to a bending configuration due to the deformation of the spring, as shown in Figure 12. To facilitate the establishment of the static model, assume that there are *n* joints in the distal segment, and there is no deformation in the proximal segment. In addition, the robot’s gravity, vibration [42], clearance, friction between cable and cable hole, and cable elastic deformation are ignored, and only external force and spring elasticity are considered to establish the static model based on the principle of minimum potential energy. Moreover, the length changes of cables *E* and *F* in the proximal segment can be not considered in the process of the static model, due to the decoupling motion between the proximal segment and distal segment.

Considering that the process of applying external forces is gradual and the total potential energy in the robot system consists of external work and elastic potential energy, the total potential energy function of the robot system can be expressed as Equation (17).

(17) $$W=W_{ext}+V_{S}+\lambda \left(\Delta S_{r}-\Delta S_{l}\right)=\frac{1}{2}\left(F_{y}\Delta y+F_{x}\Delta x\right)+\frac{1}{2}K{\left(\sum_{i=1}^{n}\Delta l_{r}\left(\psi_{i}\right)\right)}^{2}+\lambda \left(\sum_{i=1}^{n}\Delta l_{r}\left(\psi_{i}\right)-\sum_{i=1}^{n}\Delta l_{l}\left(\psi_{i}\right)\right)$$

where *W_ext_* represents the external work, *V_s_* represents the elastic potential energy, λ represents the Lagrange multiplier, and $\Delta S_{r}$, $\Delta S_{l}$ represent length changes of cables *E* and *F*. *K* is the spring stiffness. 

Therefore, the deformation angle $\Delta \psi_{i}=\left[\Delta \psi_{1},\Delta \psi_{2},\Delta \psi_{3},\cdot \cdot \cdot ,\Delta \psi_{n}\right]$ of the distal segment suffering external forces can be calculated when the potential energy is the minimum [43], which can be expressed as

(18) $$\frac{\partial W}{\partial \psi_{1}}d\psi_{1}+\frac{\partial W}{\partial \psi_{2}}d\psi_{1}+\cdot \cdot \cdot +\frac{\partial W}{\partial \psi_{n-1}}d\psi_{n-1}+\frac{\partial W}{\partial \psi_{n}}d\psi_{n}=0$$

assuming that the distal segment deforms in the *z*-*y* bending plane when the end of the distal segment is subject to external forces. The deformation of the distal end is defined as Δξ. Hence, the external work *Wext* can be calculated as

(19) $$W_{ext}=\frac{1}{2}F\cdot \Delta \xi$$

where *F* = [*F_z_*, *F_y_*] and Δξ = [Δ*z*, Δ*y*]^T^. *Fz* is the axial force along the robot, and *Fy* is the force perpendicular to the axis. When the distal segment is not subjected to external forces, the end positions *z*_0_ and *y*_0_ of the distal segment can be derived as

(20) $$\begin{array}{l} z_{0}=\left(2R+t\right)\left(\cos\frac{\psi_{0}}{2}+\cos\frac{3\psi_{0}}{2}+\ldots +\cos\frac{\left(2n-1\right)\psi_{0}}{2}\right)\\ y_{0}=\left(2R+t\right)\left(\sin\frac{\psi_{0}}{2}+\sin\frac{3\psi_{0}}{2}+\ldots +\sin\frac{\left(2n-1\right)\psi_{0}}{2}\right) \end{array}$$

where $\psi_{0}$ represents the initial bending angle. When the end of the distal segment is subject to external forces, the deformations in two directions can be derived as

(21) $$\begin{array}{l} \Delta z=\left(2R+t\right)\left(\left(\cos\left(\Delta \psi_{1}+\frac{\psi_{0}}{2}\right)+\ldots +\cos\left(\Delta \psi_{1}+\Delta \psi_{2}+\ldots +\Delta \psi_{n}+\frac{\left(2n-1\right)\psi_{0}}{2}\right)\right)-\left(\cos\frac{\psi_{0}}{2}+\ldots +\cos\frac{\left(2n-1\right)\psi_{0}}{2}\right)\right)\\ \Delta y=\left(2R+t\right)\left(\left(\sin\left(\Delta \psi_{1}+\frac{\psi_{0}}{2}\right)+\ldots +\sin\left(\Delta \psi_{1}+\Delta \psi_{2}+\ldots +\Delta \psi_{n}+\frac{\left(2n-1\right)\psi_{0}}{2}\right)\right)-\left(\sin\frac{\psi_{0}}{2}+\ldots +\sin\frac{\left(2n-1\right)\psi_{0}}{2}\right)\right) \end{array}$$

where $\Delta \psi_{1},\Delta \psi_{2},\Delta \psi_{3},\cdot \cdot \cdot ,\Delta \psi_{n}$ represent the deformation angle of distal segments, respectively. Hence, the work of external forces can be obtained by Equations (19)–(21). Assume that the spring deformation can be denoted as ΔX. When the end of the distal segment is subject to the external forces, the elastic potential energy of the spring can be expressed as

(22) $$V_{s}=\frac{1}{2}K\cdot {\left(\Delta X\right)}^{2}$$

Since cables driving the distal segment are fixed with the same driving pulley, the spring deformation is equal to the length changes of cables on both sides, which can be expressed as

(23) $$\Delta X=\sum_{i=1}^{n}\Delta l_{E}\left(\Delta \varphi_{i}\right)=\sum_{i=1}^{n}\Delta l_{F}\left(\Delta \varphi_{i}\right)$$

According to the mapping between the actuator space and joint space of the distal segment, the length changes of cables *E* and *F* can be respectively rewritten as

(24) $$\begin{array}{l} \sum_{i=1}^{n}\Delta l_{E}\left(\Delta \varphi_{i}\right)=2\sqrt{R_{1}{}^{2}+r^{2}}\sum_{i=1}^{n}\left(\sin\left(\frac{\psi }{2}+\beta \right)-\sin\left(\frac{\psi +\Delta \psi_{i}}{2}+\beta \right)\right)\\ \sum_{i=1}^{n}\Delta l_{F}\left(\Delta \varphi_{i}\right)=2\sqrt{R_{1}{}^{2}+r^{2}}\sum_{i=1}^{n}\left(-\sin\left(\frac{\psi }{2}-\beta \right)+\sin\left(\frac{\psi +\Delta \psi_{i}}{2}-\beta \right)\right) \end{array}$$

**Figure 13:**
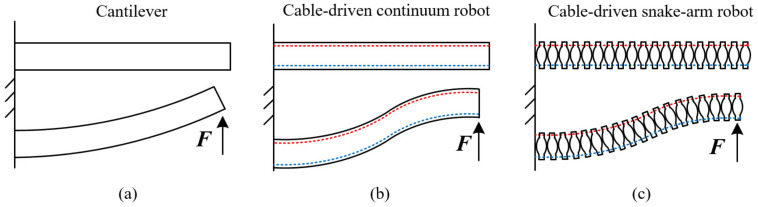
The cable constraint condition. (**a**) The bending configuration of the cantilever. (**b**) The bending configuration of the cable-driven continuum robot. (**c**) The bending configuration of the cable-driven snake-arm robot.

Hence, the elastic potential energy of the spring can be obtained by Equations (22)–(24). If there are no cable constraints, the continuum robot and snake-arm robot can be equivalent to the cantilever model. When the end of the cantilever is subject to external forces, the entire structure of the cantilever bends to one side, as shown in Figure 13a. For the cable-driven continuum robot and snake-arm robot, when the end of the robot is subject to the external force, the robot configuration will change from the straight configuration to the *S* configuration due to cable constraints, as shown in Figure 13b,c. It is necessary for establishing the static model of the cable-driven snake-arm robot to consider cable constraints [44].

For the proposed cable-driven snake-arm robot, the cable constraints can be obtained as

(25) $$\sum_{i=1}^{n}\Delta l_{l}\left(\Delta \psi_{i}\right)-\sum_{i=1}^{n}\Delta l_{r}\left(\Delta \psi_{i}\right)=4\cos\beta \sqrt{R_{1}{}^{2}+r^{2}}\sum_{i=1}^{n}\left(\sin\frac{\psi_{0}}{2}-\sin\frac{\psi_{0}+\Delta \psi_{i}}{2}\right)=0$$

Based on Equation (25), the cable constraints become more complex as the number of joints increases, which will increase the difficulty of the iterative solution. Assume that the deformation range of each joint of the robot under slight external forces is very small, and the sine Taylor expansion is used to simplify the cable constraints, which can be rewritten as

(26) $$\sum_{i=1}^{n}\Delta l_{l}\left(\Delta \varphi_{i}\right)-\sum_{i=1}^{n}\Delta l_{r}\left(\Delta \varphi_{i}\right)=-2\cos\beta \sqrt{R_{1}{}^{2}+r^{2}}\sum_{i=1}^{n}\left(\Delta \psi_{i}+ο(\Delta \psi_{i})\right)=0$$

where $-4\cos\beta \sqrt{R_{1}{}^{2}+r^{2}}\neq 0$, $ο(\Delta \psi_{i})$ represents the dimensionless. Therefore, Equation (17) can be rewritten as

(27) $$W=\frac{1}{2}\left(F_{y}\Delta y+F_{x}\Delta x\right)+\frac{1}{2}K{\left(\sum_{i=1}^{n}\Delta l_{r}\left(\Delta \psi_{i}\right)\right)}^{2}+\lambda \left(\Delta \psi_{1}+\Delta \psi_{2}+\cdot \cdot \cdot +\Delta \psi_{n}\right)$$

### 4.2. Solutions

**Figure 14:**
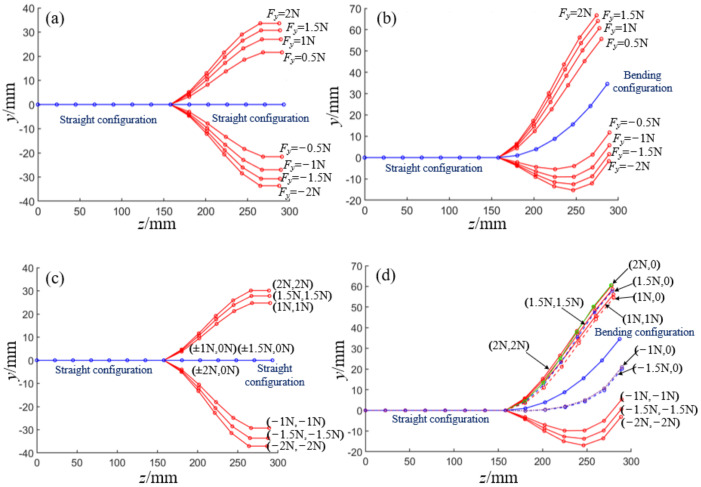
The equilibrium conformations of the distal segment under different external forces. (**a**) The equilibrium conformations of the initial distal segment under different *y*-direction forces. (**b**) The equilibrium conformations of the bending distal segment under different *y*-direction forces. (**c**) The equilibrium conformations of the initial distal segment under different two-directions forces. (**d**) The equilibrium conformations of the bending distal segment under different two-direction forces.

In this section, the deformation angles of each joint in the distal segment under different external forces, different *h_c_*_,_ and different springs are solved iteratively by MATLAB when the potential energy of the robot system is minimum. Given that *K* = 600 N/m and *h*_3_ = 11 mm, the equilibrium conformation of the distal segment in the straight configuration and bending configuration is shown in Figure 14a,b when *F_y_* = ±2 N, ±1 N, ±0.75 N, ±0.5 N, and ±0.25 N, respectively. The end displacements of the distal segment and the deformation angles of each joint increase as the magnitude of *F_y_* increases.

**Figure 15:**
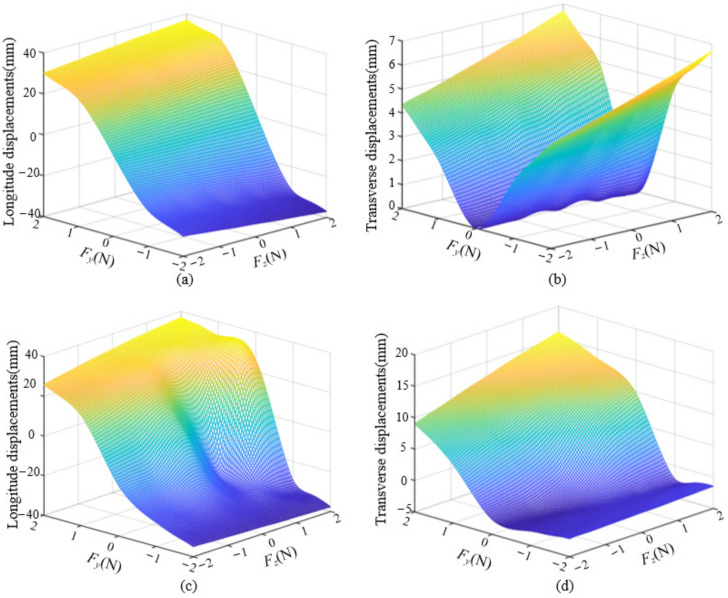
The longitudinal and transverse displacements of the end under external forces. (**a**) The relationship among the longitude displacements and two-directions in the initial configuration. (**b**) The relationship among the transverse displacements and two-directions in the initial configuration. (**c**) The relationship among the longitude displacements and two-directions.in the bending configuration. (**d**) The relationship among the transverse displacements and two-directions.in the bending configuration.

In addition, when the end is subjected to forces *F_y_* and *F_z_* at the same time, and ***F*** = [*F_z_*, *F_y_*] = {[−2 N, −2 N], [−2 N, 0 N], [−1 N, −1 N], [−1 N, 0 N], [2 N, 2 N], [2 N, 0 N], [1 N, 1 N], [1 N, 0 N]}, the equilibrium conformation of the distal segment in straight configuration and bending configuration is shown in Figure 14c,d, respectively. The configuration of the distal segment in the straight configuration does not change when only *F_z_* loads on the end, while the configuration of the distal segment in the bending configuration will change obviously when *F_y_* and *F_z_* load on the distal end. When the distal segment of the robot is in a straight configuration and its distal end is subjected to forces from two directions, the longitudinal and transverse displacements of the end are shown in Figure 15a,b, respectively. When the distal segment of the robot is in an even bending configuration and its distal end is subjected to forces from two directions, the longitudinal and transverse displacements of the end are shown in Figure 15c,d, respectively. When the spring stiffness and the initial joint height are given, the longitudinal and transverse end displacements of the distal segment of the robot will increase with the increase of the external forces.

**Figure 16:**
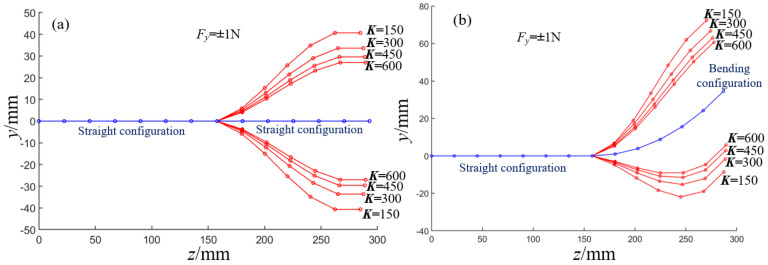
The equilibrium conformations of the distal segment under different spring stiffness. (**a**) The equilibrium conformations of the distal segment in initial configuration. (**b**) The equilibrium conformations of the distal segment in bending configuration.

**Figure 17:**
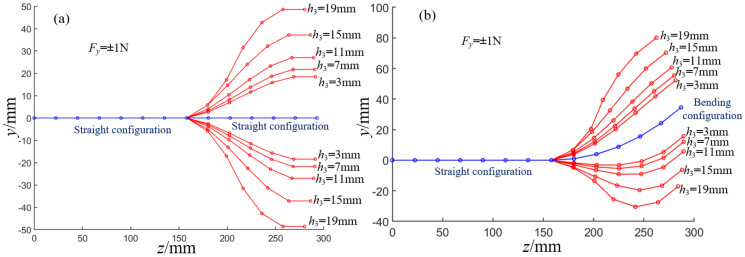
The equilibrium conformations of the distal segment under different *h*_3_. (**a**) The equilibrium conformations of the distal segment in initial configuration. (**b**) The equilibrium conformations of the distal segment in bending configuration.

According to Equation (27), the deformation angles of each joint are different if the spring stiffness *K* is different in the driving mechanism. This section only considers the force of ±1 N in the *y-*direction. When the spring stiffness *K* is equal to 150 N/m, 300 N/m, 450 N/m, 600 N/m, and 750 N/m, the equilibrium conformation of the distal segment in a straight configuration and bending configuration are shown in Figure 16a,b, respectively. According to the solution results, the larger the spring stiffness *K* is, the smaller the bending deformation angle of each joint in the distal segment is, and vice versa. In addition, when *K* is equal to 600 N/m and *h*_3_ is equal to 19 mm, 15 mm, 11 mm, 7 mm, and 3 mm, the equilibrium conformation of the distal segment in the straight configuration and bending configuration is shown in Figure 17a,b, respectively. The end displacement of the distal segment and the deformation angles of each joint decrease with the decrease of the *h*_3_. 

**Figure 18:**
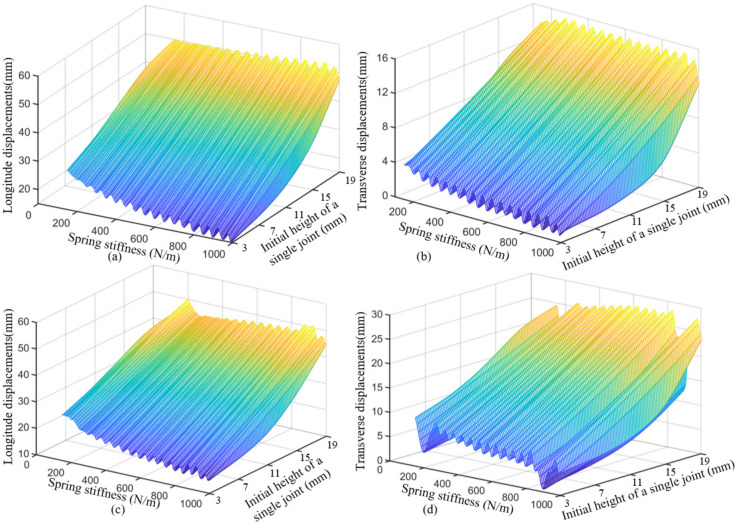
The longitudinal and transverse displacements of the end under different spring stiffness and initial joint heights. (**a**) The relationship among the longitude displacements and spring stiffness and initial height single joint in the initial configuration of the distal segment. (**b**) The relationship among the transverse displacement and spring stiffness and initial height single joint in the initial configuration of the distal segment. (**c**) The relationship among the longitude displacements and spring stiffness and initial height single joint in the bending configuration of the distal segment. (**d**) The relationship among the transverse displacement and spring stiffness and initial height single joint in the bending configuration of the distal segment.

When the distal segment of the robot is in a straight configuration and its distal end is subjected to forces from the *y* direction, the longitudinal and transverse end displacements under different spring stiffness and initial joint heights are shown in Figure 18a,b, respectively. When the distal segment of the robot is in an even bending configuration and its distal end is subjected to forces from the *y* direction, the longitudinal and transverse end displacements under different springs and initial joint heights are shown in Figure 18c,d, respectively.

When the magnitude and direction of the external forces and the spring stiffness are given, the longitudinal and transverse end displacements of the distal segment of the robot will also increase with the increase of the initial joint height. The initial joint height can be adjusted by changing the distance between the major axis and the upper surface of the lower disk. If the distance between the major axis and the upper surface of the lower disk increases, the initial joint height will decrease, and vice versa. When the magnitude and direction of the external forces and the initial joint height are given, the longitudinal and transverse end displacements of the distal segment of the robot will also decrease with the increase of the spring stiffness.

## 5. Experiments and Discussion

In this section, the prototype of the cable-driven snake-arm robot is developed. In addition, the multi-DOF bending motion of the robot is tested, and the non-existence of passive compliance and the validity of the static model are verified by load experiments. Finally, the errors analysis between experiment and simulation is discussed in detail.

### 5.1. Robot Prototype

**Figure 19:**
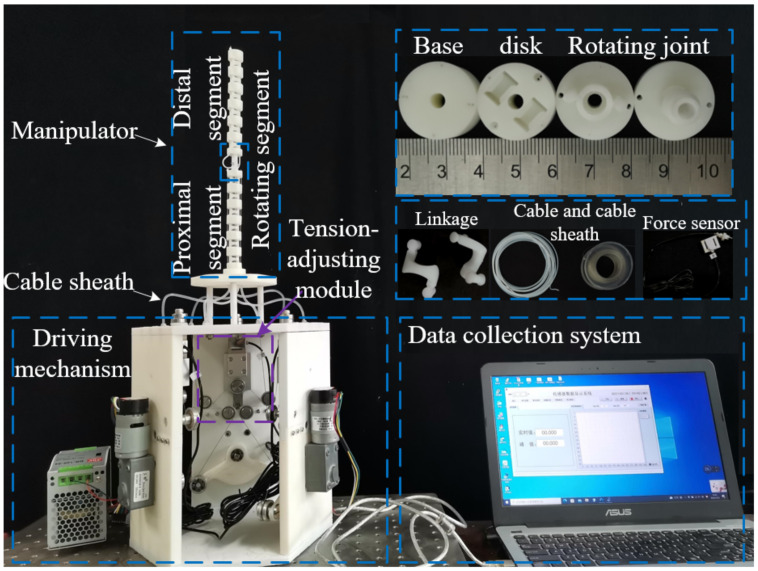
The prototype of the cable-driven snake-arm robot.

To verify the design feasibility of the cable-driven snake-arm robot based on the anti-parallelogram mechanism, a robot prototype was built in this paper, as shown in Figure 19, which mainly includes the proximal segment, the rotating segment, the distal segment, and the driving mechanism.

The driving mechanism mainly consists of a guiding device, driving device, and tension-adjusting device. The cable-driven snake-arm robot is made by 3D printing. According to Figure 8, the cable tension can be adjusted by changing the position of the adjusting bolts in the tension-adjusting device; the tension value can be measured by the force sensors. The specific parameters of the equipment used in the experiment are as follows. The rated speed and torque of the motor are 10 r/min and 70 Kg·cm, respectively. The range and accuracy of the force sensor are 0–100 N and 0.03%, respectively. The total length of the snake-arm robot is 293 mm. The robot’s diameter is 20 mm. The cable diameter is 0.6 mm.

### 5.2. Free Bending Motion Experiments

**Figure 20:**
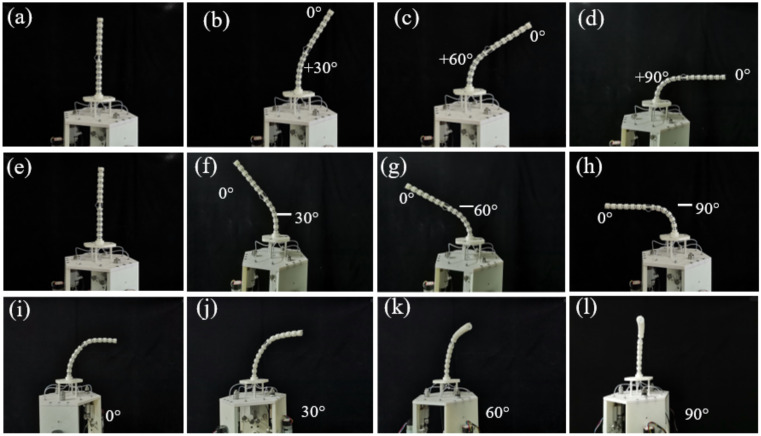
Bending configurations of the proximal segment of the robot. (**a**) The initial configuration. (**b**) The configuration with the bending angle of +30°. (**c**) The configuration with the bending angle of +60°. (**d**) The configuration with the bending angle of +90°. (**e**) The initial configuration. (**f**) The configuration with the bending angle of −30°. (**g**) The configuration with the bending angle of −60°. (**h**) The configuration with the bending angle of −90°. (**i**) The bending configuration. (**j**) The rotating 30° clockwise of driving mechanism. (**k**) The rotating 60° clockwise of driving mechanism. (**l**) The rotating 90° clockwise of driving mechanism.

This section aims to verify the performance of a cable-driven snake-arm robot with 4-DOF bending motion. For the cable-driven snake-arm robot, the cables driving the distal segment and rotating segment will not change when the proximal segment bends. Therefore, when the configurations of the rotating and distal segment remain unchanged, the bending configurations of the proximal segment of the cable-driven snake-arm robot at ±30°, ±60°, and ±90° are shown in Figure 20a–h. Therefore, the performance of bending decoupling motion is validated by the set of experiments.

When the driving mechanism rotates clockwise, taking the rotating range of [0, 90°] as an example, the configuration of the robot is shown in Figure 20i–l. 

**Figure 21:**
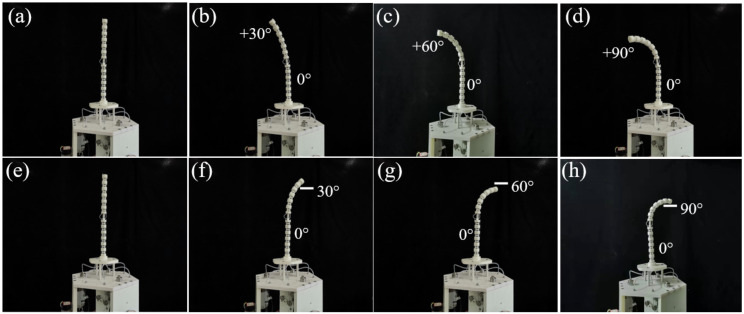
Bending configurations of the distal segment. (**a**) The initial configuration. (**b**) The configuration with the bending angle of +30°. (**c**) The configuration with the bending angle of +60°. (**d**) The configuration with the bending angle of +90°. (**e**)The initial configuration. (**f**) The configuration with the bending angle of −30°. (**g**) The configuration with the bending angle of −60°. (**h**) The configuration with the bending angle of −90°.

For the cable-driven snake-arm robot, the cables driving the proximal segment and rotating segment will not change when the distal segment bend. Thus, when the configurations of the rotating and proximal segment remain unchanged, the bending configurations of the distal segment of the cable-driven snake-arm robot at ±30°, ±60°, and ±90° are shown in Figure 21a–h. 

**Figure 22:**
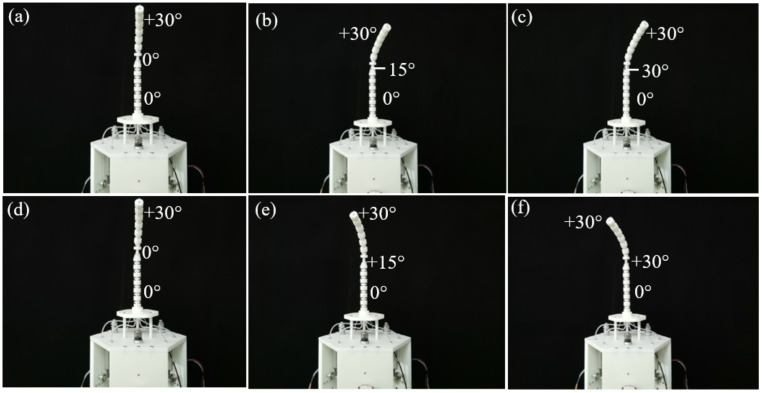
Bending configurations of the robot after rotating segment motion. (**a**) The initial configuration. (**b**) The configuration with the rotating angle of −15°. (**c**) The configuration with the rotating angle of −30°. (**d**) The initial configuration. (**e**) The configuration with the rotating angle of +15°. (**f**) The configuration with the rotating angle of +30°.

To observe the rotating motion of the rotating segment, the distal segment remains the bending configuration, while the proximal segment remains the straight configuration. Taking the rotating range of [−30°, 30°] as an example, configurations of the rotating segment clockwise and counterclockwise are measured to validate the rotating motion, as shown in Figure 22a–e.

**Figure 23:**
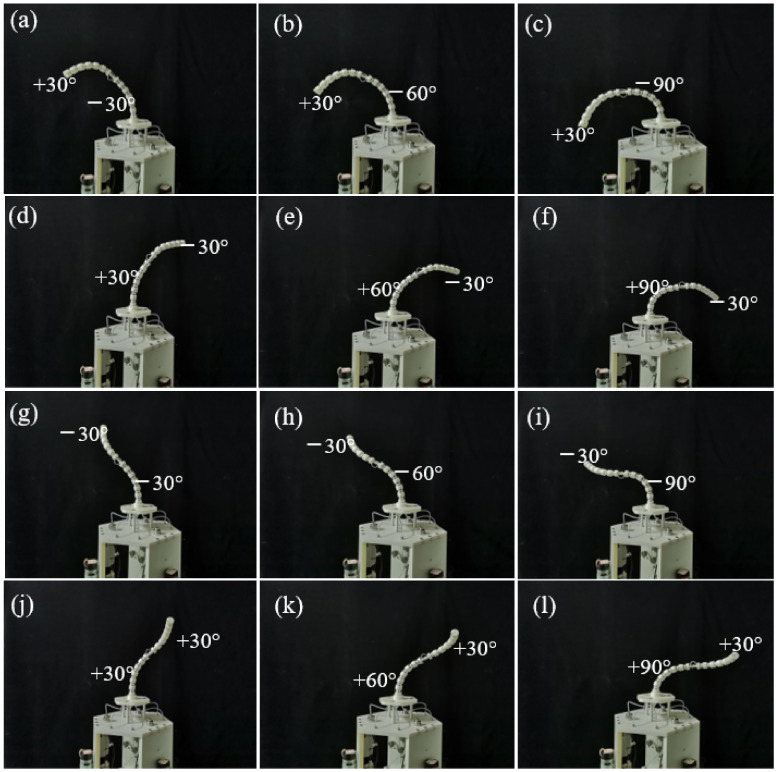
Multi-DOF bending motion test of a cable-driven snake-arm robot. (**a**) The configuration with the bending angle −30° of the proximal segment and the bending angle +30° of the distal segment. (**b**) The configuration with the bending angle −60° of the proximal segment and the bending angle +30° of the distal segment. (**c**) The configuration with the bending angle −90° of the proximal segment and the bending angle +30° of the distal segment. (**d**) The configuration with the bending angle +30° of the proximal segment and the bending angle −30° of the distal segment. (**e**) The configuration with the bending angle +60° of the proximal segment and the bending angle −30° of the distal segment. (**f**) The configuration with the bending angle +90° of the proximal segment and the bending angle −30° of the distal segment. (**g**) The configuration with the bending angle −30° of the proximal segment and the bending angle −30° of the distal segment. (**h**) The configuration with the bending angle −60° of the proximal segment and the bending angle −30° of the distal segment. (**i**) The configuration with the bending angle −90° of the proximal segment and the bending angle −30° of the distal segment. (**j**) The configuration with the bending angle +30° of the proximal segment and the bending angle +30° of the distal segment. (**k**) The configuration with the bending angle +60° of the proximal segment and the bending angle +30° of the distal segment. (**l**) The configuration with the bending angle +90° of the proximal segment and the bending angle +30° of the distal segment.

To verify the multi-DOF bending performance of the robot, the bending configurations of the distal and proximal segment of the cable-driven snake-arm robot are shown in Figure 23a–l.

### 5.3. The Load Experiments of the Proximal Segment

**Figure 24:**
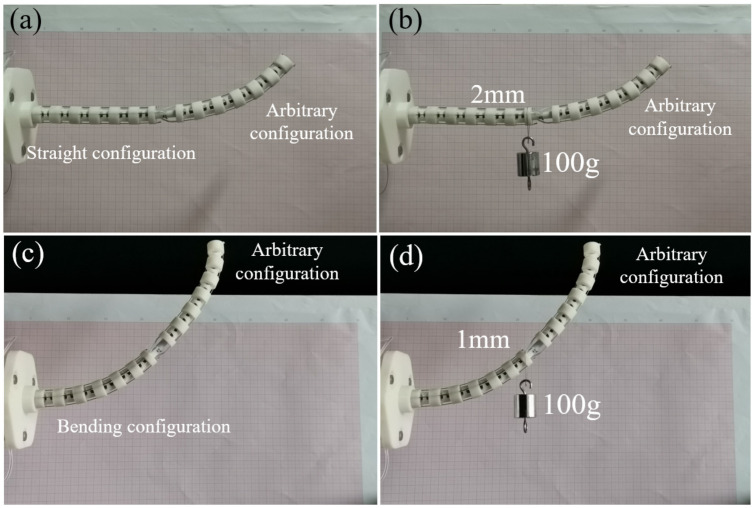
The 1 N load experiment on the end of the proximal segment. (**a**) The initial configuration of the proximal segment and the bending configuration of the distal segment. (**b**) 1N load experiment on the end in the straight configuration. (**c**) The bending configuration of the proximal segment and the bending configuration of the distal segment. (**d**) 1N load experiment on the end in the bending configuration.

To better measure the end displacements, a set of load experiments were performed in the bending plane of the distal and proximal segments. The 1 N load was applied on the straight and bending configurations of the proximal segment to verify that the cable-driven snake-arm robot does not have passive compliance, as shown in Figure 24. It means that there is no obvious S configuration or any other non-uniform configuration when the robot is subject to the external force and its inputs remain the same [6]. Therefore, the proximal segment of the cable-driven snake-arm robot is free of passive compliance.

### 5.4. The Load Experiments of the Distal Segment

**Figure 25:**
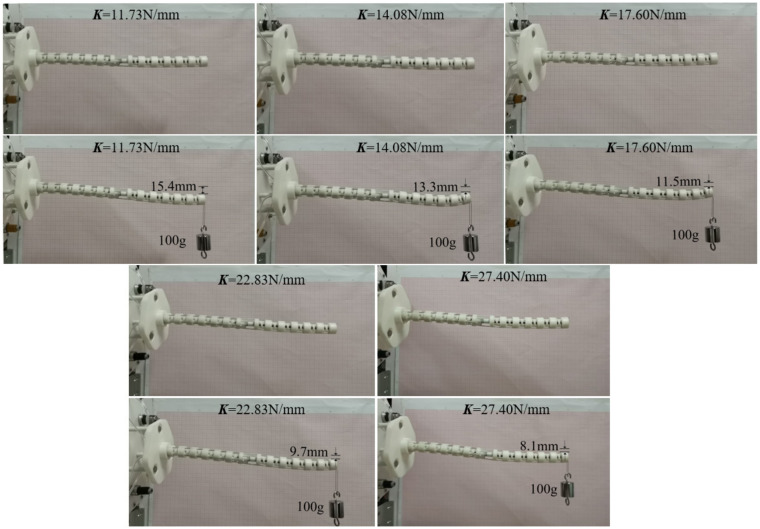
The 1 N load experiments on the distal segment with the straight configuration.

**Figure 26:**
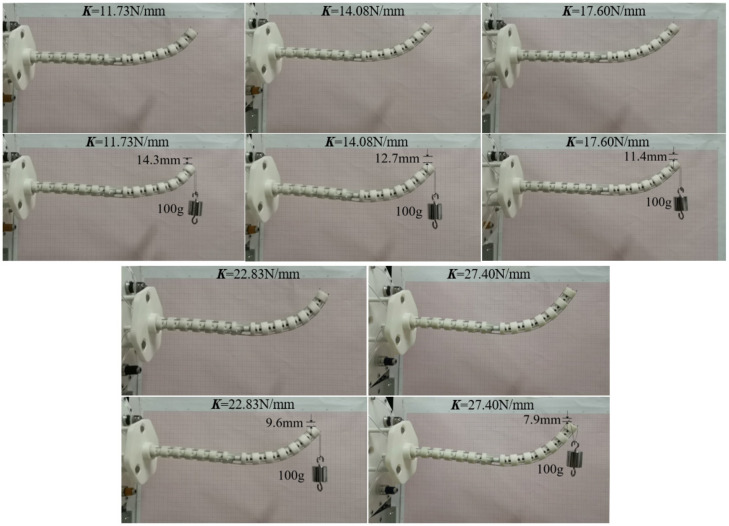
The 1 N load experiments on the distal segment with the bending configuration.

**Figure 27:**
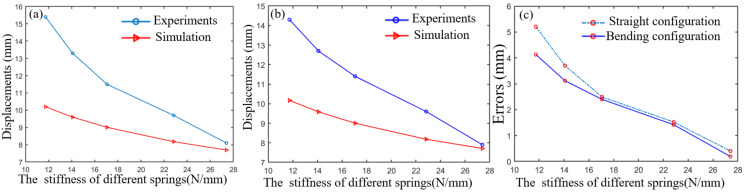
The displacement errors analysis of load experiences and simulation results. (**a**) The relationship between the longitude displacement and the stiffness of different springs in the straight configuration. (**b**) The relationship between the longitude displacement and the stiffness of different springs in the bending configuration. (**c**) The relationship between the longitude displacement and the stiffness of different springs in the bending configuration.

This section verifies the correctness of the static model of the distal segment by the longitudinal end displacements in different spring stiffness. The rotating segment is always at the initial position to better measure the end displacement of the distal segment under the external forces. When the proximal segment does not bend, 1 N load is applied to the straight configuration and bending configuration of the distal segment. To conveniently validate the static model, the end longitudinal displacements of the distal segment in the straight configuration and bending configuration in different spring stiffness are measured in Figure 25 and Figure 26. The error analysis between the simulation and experiments are shown in the Figure 27.

### 5.5. Discussion

From Figure 24, the proximal segment will still have a small end longitudinal displacement when subjected to the end force due to the gap between the cables, cable holes, and gravity. The longitudinal end displacement is less than 3 mm when the proximal segment is the straight configuration under the 1 N load, while the end displacement is less than 1 mm when the proximal segment is the bending configuration under the 1 N load. Therefore, an obvious S configuration does not appear at the proximal segment, which means that the proximal segment of the robot is still controllable when subjected to tiny external forces.

For the distal segment, the main factors for the error are as follows. Firstly, it is difficult to guarantee the absolute straight configuration of the initial angle of the distal and proximal segment in the experiment. When the initial angles are not non-zero in the straight configuration, the errors between experiments and simulations will become larger under the external forces acting on the end of the distal segment.

Secondly, there are measurement errors caused by photographic measurement. One of the future works is to improve the precision of the cable-driven snake-arm robot prototype by adopting high-precision calibration techniques to reduce the error values. 

Thirdly, the gap between the cables and the cable holes will also make the error larger. In the assembly process of the robot, the gap between the cables and the cable holes will inevitably exist. If the gap between the cables and the cable holes is too large, the error will increase when the end of the distal segment is subject to external forces. If the gap is too small, the friction will increase to make the motion performance of the robot decrease. Therefore, the tradeoff between the error variation caused by clearance and the motion performance will be the research point in the next stage.

Finally, when the load is applied to the end of the distal segment, the proximal segment will produce a deformation similar to that of the cantilever beam, which makes the displacement error increase. Therefore, the precision of the cable-driven snake-arm robot prototype can be improved by adopting high-precision manufacture, assembly, and straight configuration calibration techniques, which shows the validity of the static model to a certain extent [45]. 

## 6. Conclusions

Based on the anti-parallelogram mechanism, an approximate cylindrical rolling joint is proposed to develop the cable-driven snake-arm robot without passive compliance. The kinematics of the robot were simplified through the bending decoupling motion of multiple segments. Based on the principle of minimum potential energy, the static model of the robot was established. Furthermore, the simplified cable constraints in the static model were proposed through Taylor expansion, which facilitates the equilibrium conformation analysis of the robot under different external forces, different springs, and different initial heights. The prototype of the cable-driven snake-arm robot was built, and the multi-DOF bending performance and terminal load capacity of the robot were tested. The longitudinal end displacement was less than 3 mm when the proximal segment was the straight configuration under the 1 N load, while the displacement was less than 1 mm when the proximal segment was the bending configuration under the 1 N load. For the distal segment, the error between the theoretical displacement and experimental displacement was less than 5.5 mm. The results verified that the proposed cable-driven snake-arm robot can avoid passive compliance as well as the validity of the robot static model.

In future work, the specific application scenes will be established to further validate the cable-driven snake-arm robot with multiple segments. The robot’s gravity, clearance, vibration, friction between the cable and cable holes, cable elastic deformation, and dynamics analysis will be considered to evaluate the equilibrium conformation of the cable-driven snake-arm robot. In addition, the further miniaturization design of cable-driven snake-arm robots will be realized for minimally invasive surgery.

## Figures and Tables

**Table 1:** The main specific parameters of the robot.

Symbols	Descriptions	Values
*h_c_*	The initial distance between the major axes of adjacent ellipses	15 mm
*r*	Cable distribution circle radius	9 mm
*R*	Cylindrical rolling radius	10.5 mm
*w_c_*	The distance between the two focuses of the ellipse	10 mm
*ψ*	The angle of joint bending	[−π/9, π/9]
*h*_0_	The distance between the circle center and the upper surface of the lower disk	3 mm
*h*_1_	The distance between the major axis and the upper surface of the lower disk	2.0 mm
*h*_2_	The thickness of a single disk	11.5 mm
*h*_3_	The initial height of the single joint	11.0 mm
*h*_4_	The distance between the circle center and the upper surface of the lower disk	5 mm
